# Survival benefit of re-irradiation in esophageal Cancer patients with Locoregional recurrence: a propensity score-matched analysis

**DOI:** 10.1186/s13014-018-1122-y

**Published:** 2018-09-10

**Authors:** Liang Hong, Yun-xia Huang, Qing-yang Zhuang, Xue-qing Zhang, Li-rui Tang, Kai-xin Du, Xiao-yi Lin, Bu-hong Zheng, Shao-li Cai, Jun-xin Wu, Jin-luan Li

**Affiliations:** 1 Department of Radiation Oncology, Fujian Medical University Cancer Hospital, Fujian Cancer Hospital, Fuzhou, 350014 China; 2 Department of Radiation Oncology, Xiamen Humanity Hospital, Xiamen, China; 3 Biomedical Research Center of South China, Fujian Normal University, Fuzhou, China

**Keywords:** Esophageal squamous cell carcinoma, Locoregional recurrence, Re-irradiation, Propensity score-matched analysis, Overall survival

## Abstract

**Background:**

To investigate the treatment failure pattern and factors influencing locoregional recurrence of esophageal squamous cell carcinoma (ESCC) and examine patient survival with re-irradiation (re-RT) after primary radiotherapy.

**Methods:**

We retrospectively analyzed 87 ESCC patients treated initially with radiotherapy. Failure patterns were classified into regional lymph node recurrence only (LN) and primary failure with/without regional lymph node recurrence (PF). Patients received either re-RT or other treatments (non-re-RT group). Baseline covariates were balanced by a propensity score model. Overall survival (OS) and toxicities were assessed as outcomes.

**Results:**

The median follow-up time was 87 months. Thirty-nine patients received re-RT. Failure pattern and re-RT were independent prognostic factors for OS (*P* = 0.040 and 0.015) by Cox multivariate analysis. Re-RT with concomitant chemotherapy showed no survival benefit over re-RT alone (*P* = 0.70). No differences in characteristics were found between the groups by Chi-square tests after propensity score matching. The Cox model showed that failure pattern and re-RT were prognostic factors with hazard ratios (HR) of 0.319 (*P* = 0.025) and 0.375 (*P* = 0.002), respectively, in the matched cohort. Significant differences in OS were observed according to failure pattern (*P* = 0.004) and re-RT (*P* < 0.001). In the re-RT and non-re-RT groups, 9.09% and 3.03% of patients experienced tracheoesophageal fistulas, and 15.15% and 3.03% of patients developed pericardial/pleural effusion, respectively (*P* > 0.05). The incidence of radiation pneumonitis was higher in the re-RT group (24.24% vs. 6.06%, *P* = 0.039), but no cases of pneumonia-related death occurred.

**Conclusions:**

Re-RT improved long-term survival in patients with locoregional recurrent ESCC. Despite a high incidence of radiation pneumonitis, toxicities were tolerable.

## Background

Locoregional recurrence is the most common mode of failure in esophageal cancer treated initially with chemoradiotherapy (CRT) and/or surgery [1]. The local recurrence rate after definitive CRT has ranged from 40 to 60% with a low 5-year survival rate upon recurrence [2, 3]. To date, there is no consensus regarding a curative treatment, leaving limited treatment options for patients with locoregional recurrence esophageal squamous cell carcinoma (ESCC) after CRT.

Chemotherapy is preferred as a systemic treatment for multiple-site recurrence or distant metastasis, whereas definitive local therapy is suitable for locoregional recurrent ESCC with the goal of improving prognosis. Although salvage surgery has curative potential, studies have reported high rates of pulmonary complications (17–30%), anastomotic leakage (17–39%), intensive care unit admission (17–22%), and postoperative mortality (3–15%) with salvage surgery for locoregional recurrent ESCC after definitive CRT [4, 5]. These limit the number of patients who are candidates for salvage surgery.

Advancements in radiotherapy have allowed conformal radiation dose distribution with delivery of incremental doses to tumors and a minimal dose to adjacent critical structures. Re-irradiation has shown satisfactory clinical outcome in certain recurrent tumors such as lung cancer, head and neck cancer, high-grade glioma, and rectal cancer [6–11]. In the present study, we evaluated the clinical prognostic factors associated with overall survival (OS) in recurrent ESCC. Propensity score-matched (PSM) analysis was applied to assess clinical outcomes and toxicities of re-RT for locoregional recurrent ESCC to correct for the baseline covariates.

## Methods

### Patients

In the current study, we retrospectively examined 87 consecutive ESCC patients with locoregional recurrence who were admitted to Fujian Cancer Hospital between June 2000 and June 2014. All included patients met the following criteria: a) pathological confirmation of primary ESCC at initial diagnosis; b) a history of initial radiation; c) histological and/or PET-CT confirmation of locoregional recurrence including regional lymph node recurrence only (LN) or primary failure with/without regional lymph node recurrence (PF); d) no evidence of esophageal perforation or ulcer; and e) adequate liver, kidney, and bone marrow functioning with a Karnofsky performance status (KPS) score ≥ 70. The exclusion criteria were as follows: a) history of other malignancies; b) distant metastases; and c) confirmation of recurrence within 3 months of initial treatment.

Clinical staging at first diagnosis was determined by chest computed tomography (CT) and barium esophagram and/or endoscopic ultrasound (EUS). Re-staging of initial ESCC was done according to the 8th edition of American Joint Committee on Cancer (AJCC). The current study was approved by the Ethics Committee of Fujian Medical University Cancer Hospital, Fuzhou, China (KT2018–006-01). Because this was a retrospective study involving patient medical records, the requirement of patients’ consent was waived.

### Treatment

For initial treatment, 11 (12.6%) patients received radical resection with adjuvant radio (chemo) therapy (median dose = 52 Gy, range 40–56 Gy). Thirty-nine (44.8%) and 37 (42.6%) patients received CRT and RT, respectively. Among the 43 (49.4%) cases initially treated with chemotherapy (median of 3 cycles, range 1–6 cycles), 30 (34.5%) received cisplatin and paclitaxel, whereas the remaining 13 (14.9%) received cisplatin or oxaliplatin.

Patients were treated with 6- or 10-MV linear accelerators for initial radiotherapy with 1.8–2.2 Gy/fraction and 5 fractions/week. Initial RT was conventional two-dimensional (36.8%) or conformal three-dimensional (63.2%) RT with a median dose of 62 Gy (range 40–76 Gy). The median dose of re-RT was 50 Gy (ranged 21–70 Gy) with 2Gy (range 1.8–4 Gy) per fraction. Intensity-modulated RT (56.4%; 22/39) and conformal three-dimensional RT (43.6%; 17/39) were employed for re-RT. Among the 39 patients treated with re-RT, 19 patients (48.7%) received concomitant chemotherapy, of which 6 received cisplatin, 4 received 5-flurouracil (5-FU), and 9 received cisplatin combined with 5-FU. The remaining 20 (51.3%) patients received RT alone.

The biological effectiveness of radiation schedule was calculated by the biologically effective dose (BED) formula: BED = n × d (1 + d/(α/β)), d for the dose per fraction (Gy) and n for the number of fractions. Assuming an α/β ratio of 10 Gy for ESCC (BED_10_) [12]. For re-RT patients, the cumulative dose was calculated.

For patients without re-RT, 7 (8.0%) patients received chemotherapy alone with cisplatin combined with 5-FU, whereas 3 (3.4%) patients underwent salvage total esophagectomy with gastric pull-through. The remaining 38 (43.7%) patients received supportive care including esophageal stenting, dilation or percutaneous endoscopic gastrostomy to relieve dysphagia.

### Follow-up

The primary endpoint was OS, which was defined as the time duration from recurrence diagnosis to death or last follow-up. The recurrence-free interval (RFI) was defined as the time interval from the end of initial treatment to the recurrence diagnosis. According to the National Cancer Institute Common Terminology Criteria for Adverse Events (CTCAE) version 4.0, toxicities recorded in the patients’ medical records were retrospectively graded [13]. Tracheoesophageal fistula (TEF), pericardial/pleural effusion, and radiation pneumonitis (RP) were recorded.

### Statistical analysis

All statistical tests were performed using SPSS version 22.0 (IBM Corporation, Armonk, NY, USA). The propensity score matching ratio was set to 1:1 to minimize differences due to age, gender, primary tumor location, and initial clinical stage. Chi-square (χ^2^) and Fisher’s exact tests were applied to compare unmatched background factors. Survival curves were constructed and compared by the Kaplan-Meier method and log-rank tests. The Cox regression model was employed for the univariate analysis and multivariate analysis. *P*-values < 0.05 were considered statistically significant.

## Results

### Patient characteristics

The patient characteristics are summarized in Table 1. The median age was 62 years (range 39–86 years), and the study population included 65 (74.7%) males and 22 (25.3%) females. Considering KPS at recurrence diagnosis, 32 (36.8%) of patients were 70–80, while 55 (63.2%) were ≥ 80. Eight (9.2%) patients had stage I disease, 18 (20.7%) had stage II, and 61 (70.1%) had stage III at the initial diagnosis. The primary tumor location was the upper thoracic esophagus in 34 (39.1%) patients and the middle and lower thoracic esophagus in 53 (60.9%) patients. The median RFI was 16 months (range 3–168 months), and the RFI was ≤12 months in 38 (43.7%) patients and > 12 months in 49 (56.3%) patients. The failure pattern of 62 (71.3%) patients was primary recurrence, 14 (16.1%) cases of regional LN recurrence alone and 11 (12.6%) cases of combined sites. All patients were divided into two groups, 14 patients with regional LN recurrence and the remaining 73 patients with PF. For re-RT patients, 36 of 39 cases received in-field re-irradiation, while the other three cases experienced out-field locoregional failure.

**Table 1:** Characteristics of 87 patients with locoregional recurrent esophageal cancer

Variables	Number	Percent
Age (years)		
< 65	58	66.7
≥ 65	29	33.3
KPS		
70–80	32	36.8
>80	55	63.2
Gender		
Male	65	74.7
Female	22	25.3
Smoking		
Yes	31	35.6
No	56	64.4
Alcohol consumption		
Yes	15	17.2
No	72	82.8
Primary tumor location		
Upper thoracic	34	39.1
Middle and lower thoracic	53	60.9
Initial clinical stage		
I + II	23	32.9
III	47	67.1
Initial treatment		
Surgery + adjuvant radio(chemo)therapy	11	12.6
Definitive chemoradiotherapy	39	44.8
Definitive radiotherapy	37	42.6
Pattern of recurrence		
Regional lymph node recurrence only	14	16.1
Local failure	62	71.3
Both	11	12.6
Radiation dose in the initial treatment (Gy)		
≤ 50	12	13.8
> 50	75	86.2
Recurrence-free interval (months)		
≤ 12	38	43.7
> 12	49	56.3
Chemotherapy after recurrence		
Yes	26	29.9
No	61	70.1
Re-RT after recurrence		
Yes	39	44.8
No	48	55.2
Treatment moditily after recurrence		
Re-RT only	20	23.0
CRT only	7	8.0
Re-RT concomitant chemotherapy	19	21.8
Best supportive care	39	43.7
Salvage total esophagectomy	3	3.4

*Abbreviation*: *Re-RT* Re-irradiation, *KPS* Karnofsky performance status

For re-RT patients, the median BED_10_ of 74.11 Gy (range 48–86.32 Gy) and 60 Gy (range 25.41–84.87 Gy) were delivered in the initial radiation and re-RT, respectively. The median cumulative BED_10_ was 135.53 Gy (range 96–168 Gy). The median Dmax of spinal cord was 25 Gy (range 9–39 Gy), the median V20 of the total lung and V30 of the heart was 10% (range 0–24%) and 9% (range 0–25%), respectively.

### OS for the total study population

**Fig. 1:**
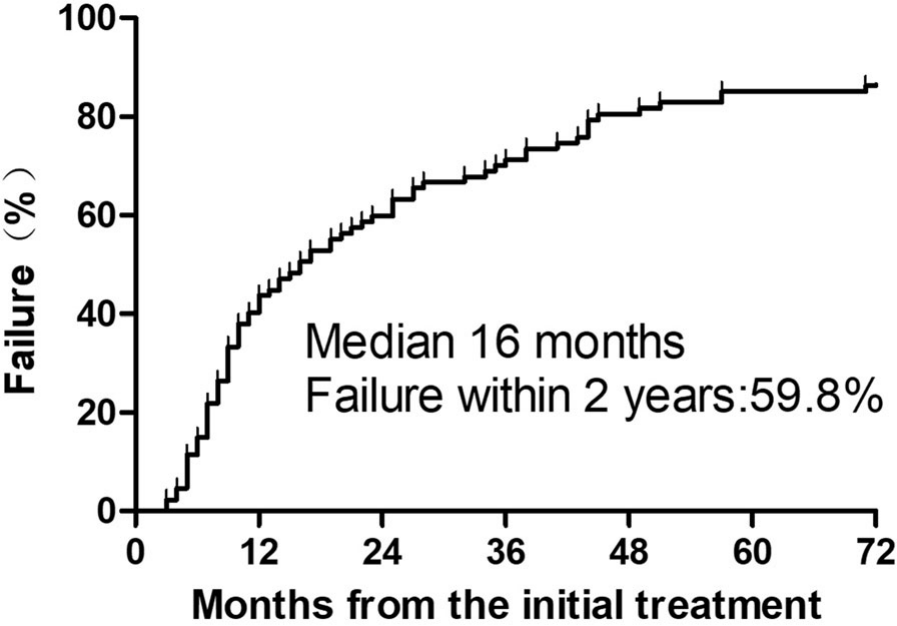
Time to locoregional recurrence after initial treatment for 87 ESCC patients

The median follow-up was 87 months (range 2–206 months). The follow-up rate was 96.6% (84/87). One patient without re-RT and 7 patients with re-RT remained alive at the last follow-up. For re-RT, 82.1% (32/39) patients were dead at last follow up, among which, 84.4% (27/32) patients were cancer-related death. After re-RT, 89.7% (35/39) suffered from failure, with 24 (68.6%) cases of distant metastasis alone, 5 (14.3%) cases of local failure alone and 6 (17.1%) cases of both. The median survival time (MST) was 10 months (range 1–85 months). The median RFI was 16 months (range 3–168 months). Fifty-two (59.8%) patients were diagnosed with recurrence within 2 years after initial treatment (Fig. 1).

### Propensity score matching and χ^2^ tests

Significant differences in the clinical stage of initial cancer were observed for patients with re-RT (*n* = 39) and without re-RT (*n* = 48) before matching (*P* = 0.003) (Table 2). A nearest neighbor and 1:1 matching algorithm was applied within a default caliper (0.2) [14]. After matching, baseline covariates of the clinicopathological characteristics were corrected, with characteristics being evenly distributed between the re-RT group (*n* = 33) and the non-re-RT group (*n* = 33, all *P* > 0.1).

**Table 2:** Chi-square test of re-RT and without re-RT for locoregional recurrent ESCC before and after matching

Variables	Before matching			After matching		
	With re-RT (n)	Without re-RT (n)	*P*	With re-RT (n)	Without re-RT (n)	*P*
Age (years)			1.000			1.000
< 65/≥65	26/13	32/16		21/12	21/12	
KPS			0.770			0.802
70–80/>80	15/24	17/31		13/20	14/19	
Gender			0.572			0.580
Male/Female	28/11	37/11		23/10	25/8	
Smoking			0.619			0.796
Yes/No	15/24	16/32		12/21	11/22	
Alcohol consumption			0.679			1.000
Yes/No	6/33	9/39		5/28	5/28	
Primary tumor location			0.583			0.802
Upper/Middle and lower thoracic	14/25	20/28		14/19	13/20	
Initial clinical stage			0.003			0.284
I + II/III	18/21	8/40		12/21	8/25	
Surgery in the initial treatment			0.488			0.392
Yes/No	6/33	5/43		2/31	4/29	
Chemotherapy in the initial treatment			0.582			0.806
Yes/No	18/21	25/23		16/17	17/16	
Radiation dose in the initial treatment (Gy)			0.101			0.213*
≤ 50/> 50	8/31	4/44		5/28	2/31	
Recurrence-free interval (months)			0.187			0.215
Median (range)	27(4163)	12.5(3168)		27(5163)	12(3144)	
≤ 12/> 12	14/25	24/24		12/21	17/16	

*Abbreviation*: *Re-RT* Re-irradiation, *KPS* Karnofsky performance status. *:Fisher's exact tests

### Cox regression analysis for overall sample

**Fig. 2:**
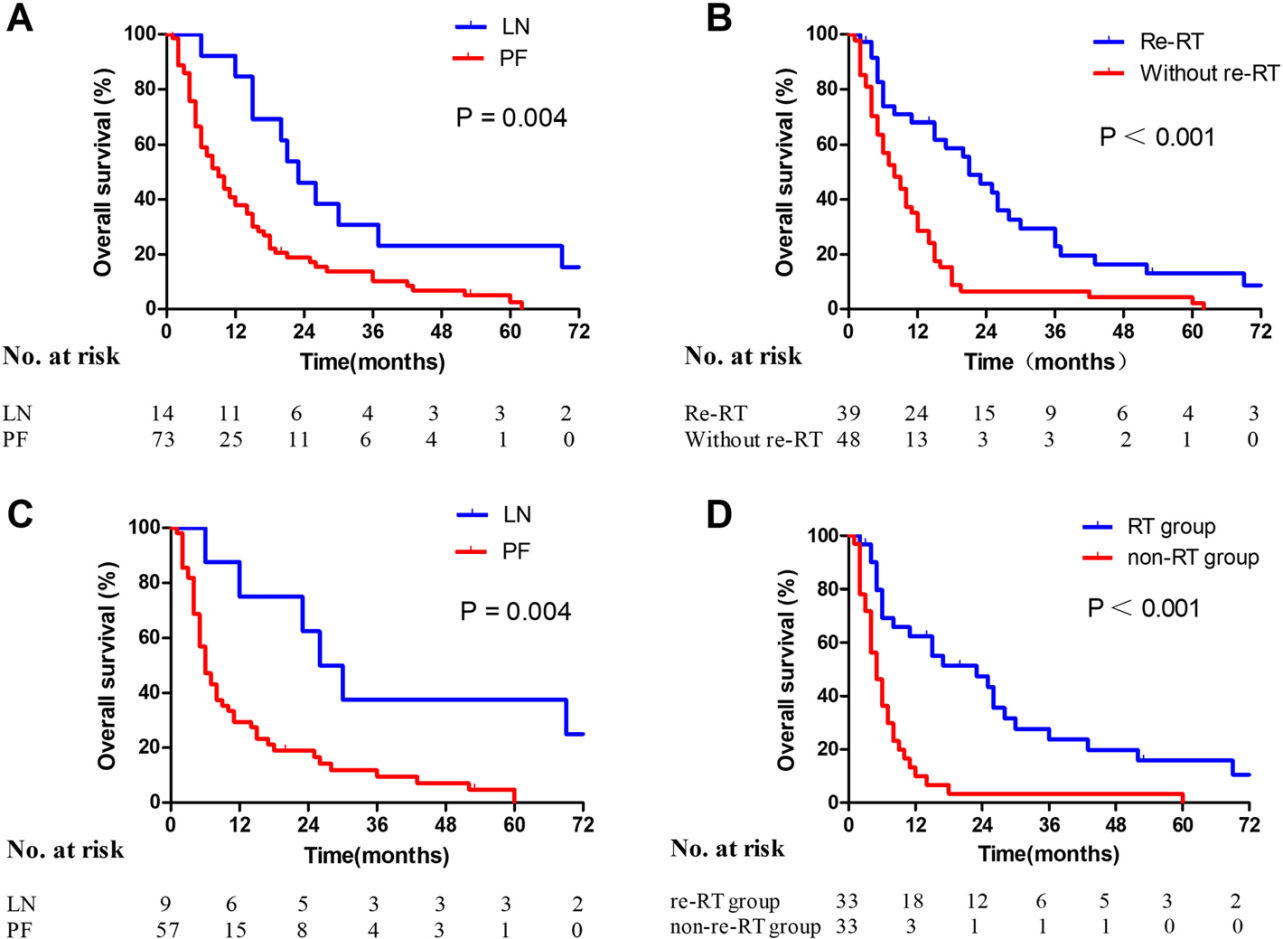
Kaplan–Meier analysis of OS according to (**a**) failure pattern (LN vs. PF, *P* = 0.004) before matching; (**b**) re-irradiation (re-RT vs. without re-RT, *P* < 0.001) before matching; (**c**) failure pattern (LN vs. PF, *P* = 0.004) after matching; and (**d**) re-irradiation (re-RT group vs. non-re-RT group, *P* < 0.001) after matching

The results of univariate and multivariate analyses for OS are summarized in Table 3. LN recurrence alone and re-RT were associated with better OS (*P* = 0.006 and *P* < 0.001) by Cox univariate analysis. The 1-, 3-, and 5-year OS rates in the LN group were 84.62%, 30.77%, and 23.01%, respectively, and the 1-, 3-, and 5-year OS rates in the PF group were 37.86%, 10.29%, and 2.57%, respectively. The MST in the LN group was 23 months, whereas the MST in the PF group was 9 months (*P* = 0.004, Fig. 2a). The 1-, 3-, and 5-year OS rates in the re-RT group were 67.94%, 22.89%, and 13.08%, respectively, and the 1-, 3-, and 5-year OS rates for patients without re-RT were 28.52%, 6.58%, and 2.19%, respectively. Their MSTs were 21 months and 8 months, respectively (*P* < 0.001, Fig. 2b).

**Table 3:** Cox model analysis for 87 ESCC patients with locoregional recurrence before matching

Variable	n	Univariate			Multivariate		
		HR	95%CI	*P*	HR	95%CI	*P*
Age (years)							
< 65/≥65	58/29	0.786	0.467–1.322	0.364			
KPS							
70–80/>80	32/55	0.752	0.467–1.212	0.243			
Gender							
Male/Female	65/22	1.008	0.607–1.673	0.976			
Smoking							
Yes/No	31/56	1.438	0.893–2.317	0.135			
Alcohol consumption							
Yes/No	15/72	1.229	0.668–2.261	0.507			
Primary tumor location							
Upper/Middle and lower thoracic	34/53	1.241	0.779–1.977	0.364			
Clinical stage							
I + II/III	26/61	1.083	0.645–1.820	0.763	1.027	0.599–1.761	0.923
Surgery in the initial treatment							
Yes/No	11/76	1.161	0.608–2.216	0.652			
Chemotherapy in the initial treatment							
Yes/No	43/44	0.954	0.601–1.514	0.842			
Recurrence-free interval (months)							
≤12/> 12	38/49	0.884	0.558–1.402	0.601			
Pattern of recurrence							
LN/PF	14/73	0.385	0.195–0.762	0.006	0.461	0.221–0.964	0.040
Re-RT after recurrence							
Yes/No	39/48	0.392	0.239–0.642	< 0.001	0.513	0.299–0.878	0.015
Chemotherapy after recurrence							
Yes/No	26/61	0.799	0.478–1.335	0.391			
Chemotherapy for both course treatment							
Yes/No	13/74	0.540	0.274–1.063	0.074	0.520	0.257–1.051	0.069

*Abbreviations*: *HR* Hazard ratio, *95%CI* 95% confidence interval, *Re-RT* Re-irradiation, *LN* Regional lymph node recurrence only, *PF* Primary failure with/without regional lymph node recurrence, *KPS* Karnofsky performance status

**Fig. 3:**
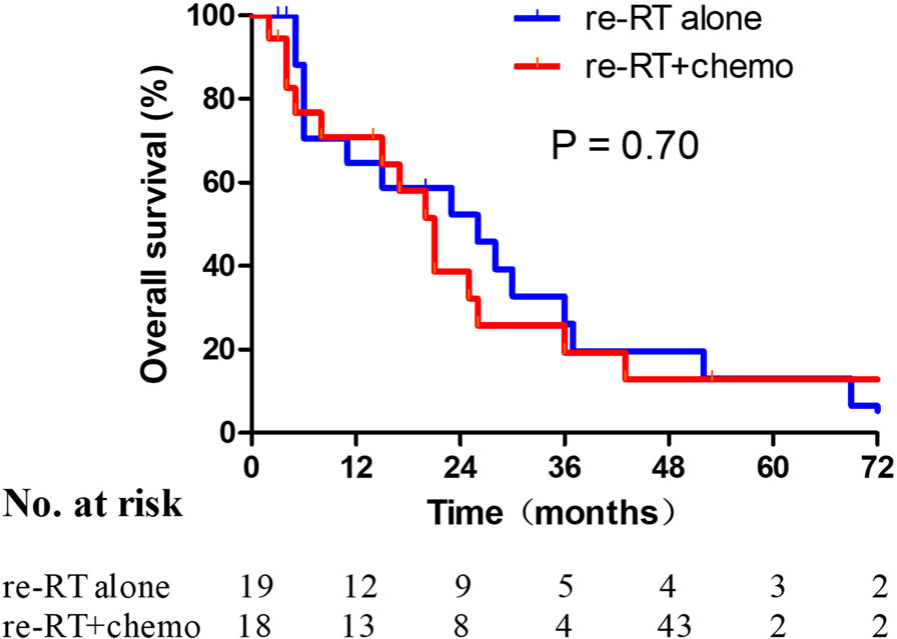
Patient survival after re-RT with or without chemotherapy

Initial clinical stage (I + II vs. III), failure pattern (LN vs. PF), re-RT (with vs. without), and chemotherapy for both courses of treatment (with vs. without) were possible prognostic factors in the Cox multivariate model. The failure pattern and re-RT were independent prognostic factors for OS (*P* = 0.040 and *P* = 0.015, respectively). However, no statistical difference in OS was observed between the re-RT alone and re-RT with concomitant chemotherapy groups (18 vs. 19, *P* = 0.70, Fig. 3) in the subgroup analysis.

### Cox regression analysis for matched cohort

In the matched cohort, failure pattern and re-RT were independently associated with OS for recurrent ESCC (*P* = 0.025 and *P* = 0.002, respectively; Table 4). For the two failure patterns (LN vs. PF), the comparative 1-, 3-, and 5-year OS rates were 75.00% vs. 29.49%*,* 37.50% vs. 9.53%, and 37.50% vs. 0%, respectively. The MSTs in the LN and PF groups were 28 months and 6 months, respectively (*P* = 0.004, Fig. 2c). For treatment with re-RT or no re-RT (with or without), the comparative 1-, 3-, and 5-year OS rates were 62.38% vs. 9.93%, 23.71% vs. 3.31%, and 15.81% vs. 0%, respectively. The MSTs in the re-RT and without re-RT groups were 23 months and 5 months, respectively (*P* < 0.001, Fig. 2d).

**Table 4:** Cox model analysis for 66 ESCC patients with locoregional recurrence after matching

Variable	n	Univariate			Multivariate		
		HR	95%CI	*P*	HR	95%CI	*P*
Age (years)							
< 65/≥65	42/24	0.736	0.407–1.330	0.310			
KPS							
70–80/>80	27/39	0.843	0.491–1.447	0.535			
Gender							
Male/Female	48/18	0.942	0.530–1.674	0.838			
Smoking							
Yes/No	23/43	1.486	0.857–2.577	0.159			
Alcohol consumption							
Yes/No	10/56	1.083	0.526–2.233	0.828			
Primary tumor location							
Upper/Middle and lower thoracic	27/39	1.265	0.733–2.183	0.398			
Clinical stage							
I + II/III	20/46	1.021	0.561–1.858	0.946	1.541	0.814–2.916	0.184
Surgery in the initial treatment							
Yes/No	6/60	2.258	0.942–5.414	0.068			
Chemotherapy in the initial treatment							
Yes/No	33/33	1.005	0.585–1.729	0.984			
Recurrence-free interval (months)							
≤12/> 12	29/37	0.739	0.433–1.262	0.268			
Pattern of recurrence							
LN/PF	9/57	0.277	0.108–0.714	0.008	0.319	0.117–0.869	0.025
Re-RT after recurrence							
Yes/No	33/33	0.299	0.167–0.535	< 0.001	0.375	0.201–0.701	0.002
Chemotherapy after recurrence							
Yes/No	20/46	0.817	0.456–1.463	0.497			
Chemotherapy for both course treatment							
Yes/No	10/56	0.621	0.291–1.323	0.217	0.710	0.323–1.562	0.395

*Abbreviations*: *HR* Hazard ratio, *95%CI* 95% confidence interval, *Re-RT* Re-irradiation, *LN* Regional lymph node recurrence alone, *PF* Primary failure with/without regional lymph node recurrence, *KPS* Karnofsky performance status

### Toxicity

In the re-RT and non-re-RT groups of the matched cohort, 9.09% (3/33) and 3.03% (1/33) of cases experienced TEF, 15.15% (5/33) and 3.03% (1/33) of cases experienced pericardial/pleural effusion (*P* = 0.613 and *P* = 0.197, respectively). The rates of grade 3 RP were 24.24% (8/33) and 6.06% (2/33) in the re-RT and non-re-RT groups, respectively (*P* = 0.039). No case of grade 5 RP was observed. The median age of the 10 patients who developed RP was 61 years (range 43–83 years). The radiation doses for primary RT in 2 patients not treated with re-RT were 63 Gy and 70 Gy. The median doses for primary RT and re-RT in the other eight patients were 62.2 Gy (range 41–64 Gy) and 50.3 Gy (range 36–60 Gy), respectively. No significant correlation was found between RP and the V20 of the total lungs in re-RT (*P* = 0.25). No treatment-related deaths were recorded.

## Discussion

Locoregional recurrence occurs frequently after primary definitive RT or multimodal therapy for ESCC. Yet, therapeutic options remain limited, and no consensus regarding the optimal treatment has been reached. Re-RT for the management of recurrent ESCC is one of the options, and in the present study, the effectiveness and toxicity of re-RT were retrospectively analyzed via PSM analysis. In the whole cohort, the failure pattern and re-RT were found to be independent prognostic factors for OS (*P* = 0.040 and *P* = 0.015, respectively), and these results were also verified in the two well-balanced groups after propensity score matching. Furthermore, significant differences in OS and MST were observed for different failure patterns (LN vs. PF, MST 28 months vs. 6 months, *P* = 0.004) as well as for re-RT (re-RT vs. non-re-RT, MST 23 months vs. 5 months, *P* < 0.001).

The current study showed that in the majority of cases (59.8%), locoregional recurrence occurred within 2 years after initial treatment. The median RFI was 16 months (range 3–168 months), which was similar to the results of Chen et al. [15]. PF was the most common (71.3%) failure pattern, followed by regional LN alone (16.1%) and both (12.6%). This distribution deviated slightly from that in a previous study by Versteijne et al., which was 57%, 14% and 29% respectively [16]. This might be attributed to differences in the pathological composition of the tumors or radiation doses given for initial treatment. Also, in the current study, failure pattern (LN vs. PF) was an independent prognostic factor for OS. PF indicated a worse OS compared to LN (*P* = 0.004, HR = 0.3754, 95% confidence interval [CI] 0.1939–0.7266), which emphasized that good control of the primary tumor plays a vital role in ESCC management.

Patients with recurrent ESCC previously treated with RT who are in good clinical condition could be selected for potentially curative treatment. Previous study had reported encouraging outcomes of re-RT for symptoms relief [17], in which 4 had complete resolution and 4 had diminished or stable symptoms among the 10 patients who presented with symptomatic disease. Moreover, Zhou et al. [18] reported that the 3-years OS for primary tumor recurrent ESCC was 21.8% with a MST of 20 months upon salvage RT group. Similarly, the 3-years OS was 22.89% among our re-RT patients with a MST of 21 months. The re-RT group had a significantly higher OS compared to the non-re-RT group in the current matched cohort (*P* < 0.001, HR = 0.2426, 95% CI 0.1294–0.4547). Yamashita et al. [19] reported a MST of 13.8 months for locoregional recurrent ESCC patients with re-RT. This inferior MST might be related to differences in the recurrent tumor location and initial treatment baseline characteristics. Salvage doses of re-irradiation should be delivered to patients with localized disease to improve local control and OS.

Concurrent CRT is the standard treatment for ESCC patients who decline or cannot tolerate surgery. However, no evidence of survival benefits from concurrent CRT was found. Concurrent CRT was shown to cause severe acute esophagitis in 15–25% of thoracic radiotherapy cases [20]. In addition, most cases of recurrent ESCC occurred in older patients for whom concurrent CRT might be sub-optimal. In the current subgroup analysis, no statistical difference in OS was found between the groups treated with re-RT alone and re-RT combined with chemotherapy (*P* = 0.70). Also, two of three cases suffered from TEF upon concurrent CRT. Thus, concurrent CRT might increase toxicity without a survival benefit.

Concerning the potentially serious complications, re-RT was performed in a small and highly selected group of patients in clinical practice. In a prospective and randomized study, which included 34 patients who received re-RT and 35 patients who received dilatation alone, 6 cases of TEF were observed in the non-re-RT group, while no case of TEF was found in the re-RT group [21]. In the current study, no statistical differences were found in the incidence of TEF (*P* = 0.613) and pericardial/pleural effusion (*P* = 0.197) between re-RT and non-re-RT groups. As reported by Yamaguchi et al. [19], advanced T stage (T3 or T4) at the recurrence diagnosis was significantly associated with grade 3 or above toxicities. This might imply that TEF might associated with tumor progression. However, the impact of repair disability for re-irradiated tissues should also be considered.

RP is another concern in thoracic re-RT. Sumita et al. [22] had retrospectively analyzed 21 lung cancer patients who underwent X-ray beam re-RT and only one grade 3 RP was observed. The incidence of grade 3 RP was 24.24% for re-RT group in our study, but even with this high incidence of RP, no pneumonia-related deaths occurred. There was no correlation between RP and the V20 of the total lungs in the present study, which might relate to the limited sample, the different initial radiation schedules and interval. In addition, Ren et al. [23] showed that both re-RT and initial-RT influenced the incidence of grade 3 or above RP. However, further studies concerning the toxicities of the OARs are required.

As a retrospective study, records for symptoms such as dysphagia, weight loss, hoarseness, and cough were not available, and thus, symptom control was not evaluated in the present study. Moreover, because this was a single-center study, the number of cases was limited due to the rarity of re-RT treatment. Therefore, the implications of the findings could be limited.

## Conclusions

Re-RT was feasible and beneficial for locoregional recurrent ESCC patients after primary RT. Compared to CRT, re-RT alone is more appropriate. Long-term survival was improved with re-RT. Despite a high incidence of RP, toxicities were tolerable.

## Abbreviations

5-FU: 5-flurouracil
AJCC: American Joint Committee on Cancer
CRT: Chemoradiotherapy
CT: Computed tomography
CTCAE: National Cancer Institute Common Terminology Criteria for Adverse Events
ESCC: Esophageal squamous cell carcinoma
EUS: Endoscopic ultrasound
HR: Hazard ratios
KPS: Karnofsky performance status
LN: Regional lymph node recurrence only
MST: Median survival time
OS: Overall survival
PF: Primary failure with/without regional lymph node recurrence
PSM: Propensity score-matched
Re-RT: Re-irradiation
RFI: Recurrence-free interval
RP: Radiation pneumonitis
TEF: Tracheoesophageal fistula
